# Metabolic disturbances due to a high-fat diet in a non-insulin-resistant animal model

**DOI:** 10.1038/nutd.2016.47

**Published:** 2017-03-13

**Authors:** L Ramalho, M N da Jornada, L C Antunes, M P Hidalgo

**Affiliations:** 1 Laboratory of Chronobiology, Hospital de Clínicas de Porto Alegre, Porto Alegre, Brazil; 2 Graduate Program in Medical Sciences: Psychiatry, Universidade Federal do Rio Grande do Sul, Porto Alegre, Brazil; 3 Graduate Program in Medicine: Medical Sciences, Universidade Federal do Rio Grande do Sul, Porto Alegre, Brazil; 4 Department of Psychiatry and Legal Medicine, School of Medicine, Universidade Federal do Rio Grande do Sul, Porto Alegre, Brazil

## Abstract

**Objective:**

Shift workers have metabolic changes more often than day workers. It is also known that night workers prefer foods high in saturated fat. Such data suggest that shift workers are prone to cardiovascular disease. Therefore, the objective of this study was to propose an animal model to test the effect of high-fat diet (HFD) based on shift workers' diet.

**Methods:**

This is an experimental study with 20 Wistar rats. Ten rats were allocated to the control group (CG) and were fed standard diet. Ten rats were allocated to the experimental group (EG) and were fed HFD (45% fat). Serum triglycerides (TG), glucose and high-density lipoprotein-cholesterol (HDL-cho) were measured 5, 10 and 15 weeks after the beginning of the study. The amount of visceral adipose tissue (VAT) was determined. Body weight was assessed weekly, and food and water intake were measured daily. Student's *t*-test was used for independent samples, and *P*<0.05 was considered significant.

**Results:**

After 15 weeks of intervention, the EG showed increased serum levels of TG (*P*=0.001) and glucose (*P*<0.001) and decreased HDL-cho (*P*<0.001) when compared with the CG. The EG showed increased VAT (*P*=0.005) and liver weight (*P*=0.01). Food intake and water intake were higher in the CG (*P*<0.001 and *P*<0.001, respectively), whereas energy intake showed no difference (*P*=0.48). No difference was found in the weight of adrenal glands (*P*=0.07) and body weight (*P*=0.63).

**Conclusions:**

The experimental diet was effective to show changes in the serum levels of glucose, TG and HDL-cho and visceral fat in spite of no change in body weight in 15 weeks.

## Introduction

Over the past few years, there has been a significant increase in the prevalence of nutrition-related diseases, such as obesity, diabetes and cardiovascular diseases. Diet quality may be one of the most important factors associated with hyperglycaemia, hypertriglyceridaemia, hypertension, decreased high-density lipoprotein-cholesterol (HDL-cho) and increased abdominal circumference.

Shift workers are particularly affected by inadequate quality of diet, mainly night workers who often have hypertriglyceridaemia and hyperglycaemia, as well as lower levels of HDL-cho when compared with day workers.^1, 2, 3, 4^

It is known that night workers, at work, prefer cold snacks containing high concentrations of saturated fat instead of hot food. This population usually keeps this pattern also during the period they are not working.^5, 6, 7^ Their diet also have several eating events per day,^8^ including high consumption of fat,^9, 10^ especially saturated fat.^6^

It is difficult to distinguish factors related to chronic diseases in human beings; therefore, experimental studies have tried to develop a standardized diet for animal models. Studies with high-fat diets (HFDs) have been conducted in animals, especially in mice and rats, to induce metabolic changes. Some of these studies have linked an HFD with hyperphagia, weight gain, increased adiposity and suppression of hepatic glucose production stimulated by insulin.^11, 12, 13^ Such conditions may lead to hyperinsulinemia and insulin resistance. These diet models often include 15–65% of fat from different sources (soybean oil, coconut oil, olive oil, fish oil, lard and vegetable shortening, with lard and vegetable shortening being commonly used by food industry) and different times of study (3–32 weeks).^14, 15, 16, 17, 18^ The type of fat chosen directly influences the biomarkers. Lard and vegetable shortening are equally rich in saturated fatty acids and monounsaturated fatty acids, therefore, they are used in studies for causing metabolic changes effectively. Fat rich in polyunsaturated fatty acids are less harmful because they prevent insulin resistance.^12, 19, 20^ Most snacks contain fat, but experimental diets focus on only one type of saturated fat, such as lard and coconut oil. A type of snack frequently consumed by shift workers is fried meat stuffed pastry (46% fat, 23.8% saturated fatty acids, 29.3% monounsaturated fatty acids, 37.8% polyunsaturated fatty acids in 100 g).^21^ Hydrogenated fats, which are commonly used by the food industry, are correlated with cardiovascular diseases, decreasing HDL-cho and increasing low-density lipoprotein-low-density lipoprotein cholesterol (LDL-cho).^22^

Based on such evidence, it is important to mimic an HFD similar to the diet consumed by shift workers in order to analyse its effects. Therefore, the objective of this study was to test the effect of an HFD on metabolism in an animal model.

## Materials and methods

### Animals

Twenty male Wistar rats from the Reproduction and Experimentation Centre of Laboratory Animals (CREAL, Centro de Reprodução e Experimentação de Animais de Laboratório; Universidade Federal do Rio Grande do Sul, Porto Alegre, Brazil) were used. The animals were 60 days old at the beginning of the study. They were housed in polycarbonate cages, five animals per cage, kept at 22±2 °C with a 12:12 h light:dark cycle (lights on at 0700 hours) according to the Guide to the Use and Care of Laboratory Animals.^23, 24^ One animal died during the experiment. The study was approved by the Ethics Committee of the Hospital de Clínicas de Porto Alegre, and it was carried out in the Animal Experimental Unit of the same institution. All procedures were performed in such a way as to minimize pain and discomfort.

### Diet

All rats were allowed *ad libitum* access to food and water. The HFD created for this study consisted of 45.5% standard chow, 22.7% lard, 22.7% vegetable shortening and 9% sucrose, whereas the standard diet (control group (CG)) consisted of 100% chow Nuvilab CR-1-Nuvital (São Paulo, Brazil). The chow diet provided 3.97 Kcal g^−1^ and the HFD provided 6.25 Kcal g^−1^, 0.18 and 2.84 Kcal g^−1^ from fat, respectively. The HFD was supplied in pellets similar to those used to offer standard diet. The HFD was prepared every 4 days and stored in a refrigerator under controlled temperature (7 °C, ±2). The amount of fatty acids in each diet is described in Table 1.^25, 26, 27^

### Experimental procedures

The rats were randomized by weight and allowed a 1-week period of adaptation to the laboratory conditions and chow diet. After this, the animals were divided into two groups of 10 animals each: experimental group (EG; receiving HFD) and control group (CG; receiving standart diet). The treatment lasted 15 weeks. Food and water intake were measured daily. Body weight was assessed weekly. Blood samples were collected in non-starved animals at baseline and at 5, 10 and 15 weeks of the treatment period for glucose, HDL-cho and triglycerides (TG) analysis. The samples were obtained from the retro-orbital plexus after sedating the animals with inhaled isoflurane (Isoforine, Cristália Produtos Químicos e Farmacêuticos Ltda., São Paulo, Brazil). At the end of the treatment, the rats were killed by decapitation to facilitate blood collection and no drugs were used before this procedure. Epididymal and retroperitoneal fats were removed from visceral adipose tissue (VAT), liver and adrenal glands and weighed using a digital scale (Marte, model AS5500c, São Paulo, Brazil). This scale was also used to determine the weight of food, water and animal's body weight. The experiment was performed one time by a non-blinded researcher.

### Metabolite determinations

Serum levels of glucose, HDL-cho and TG were evaluated. Serum from trunk blood was assayed for TG and glucose using the enzymatic colorimetric assay kits produced by Roche Diagnostics (Mannheim, Germany). Levels of HDL-cho were determined by the homogeneous enzymatic colorimetric assay kits also produced by Roche Diagnostics.

### Statistical analysis

Considering a 95% confidence level, 10 animals per group were used to detect differences between the variables. The comparison between CG and EG at baseline considered food, water and energy intake, as well as organ weight. Data were expressed as mean±s.e.m. The statistical analysis was performed using Student's *t*-test for independent samples. The analysis of CG vs EG at different time points (baseline, 5 weeks, 10 weeks, 15 weeks) was performed using two-way analysis of variance and Tukey's test (*post hoc* analysis). A *P*-value <0.05 was considered to be statistically significant. The statistical analysis was carried out using the Statistical Package for the Social Sciences, version 18.0 (SPSS Inc., Chicago, IL, USA).

## Results

We investigated two groups of rats (*n*=20). One group was fed an HFD (45.5% chow) that sought to mimic the diet of shift workers for 15 weeks. The other 10 rats were fed 100% chow (control group).

### Baseline

**Figure 1:**
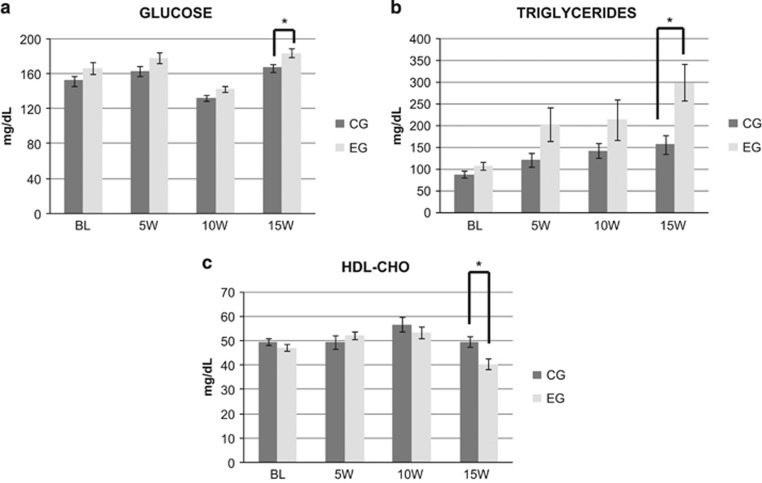
Values of serum glucose (**a**), triglycerides (**b**) and HDL-cholesterol (**c**) at baseline (BL) and at 5, 10 and 15 weeks in the CG and EG expressed as milligram per decilitre. Asterisk indicates statistical difference between CG and EG. Results are expressed as mean±s.e.m.

Data for serum glucose, TG and HDL-cho (mean±s.e.m.) are shown in Table 2 and Figure 1. No significant difference was observed between the CG and EG at baseline for body weight, serum glucose, HDL-cho and TG.

### Metabolic parameters

The comparison of serum variables (mean±s.e.m.) at baseline, 5, 10 and 15 weeks between EG and CG are presented in Figure 1. Glucose (F_19,3_=21.768; *P*<0.001), TG (F_19,3_=6.570; *P*=0.001) and HDL-cho (F_19,3_= 7,409; *P*<0.001) had statistically significant differences only at week 15. EG showed a significant increase in the levels of serum glucose (CG=166.88±3.64, EG=183.40±4.89) and TG (CG=157.33±21.51, EG=299.10±42.36) and a significant decrease in HDL-cho (CG=49.66±2.19, EG=40.40±2.22).

### Organs and tissues

**Figure 2:**
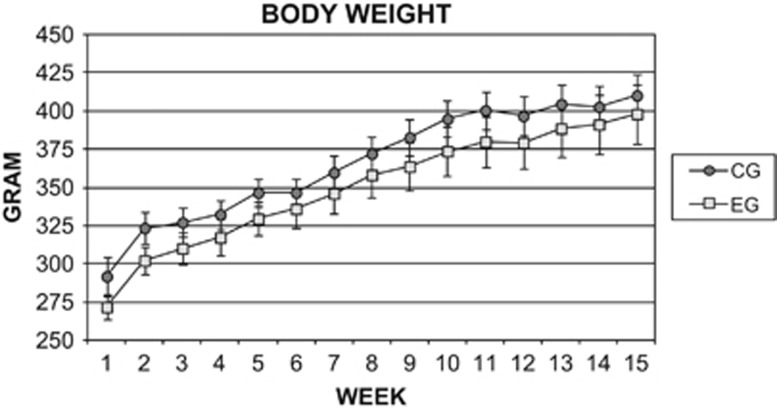
Body weight comparison during 15 weeks between the CG and EG expressed as grams. There was no statistical difference between CG and EG. Results are expressed as mean±s.e.m.

The weights of VAT, liver and adrenal glands (mean±s.e.m.), as well as body weights of the CG and EG are compared in Table 2 and Figure 2. VAT and liver weights of the EG were found to be significantly higher than in the CG. No difference was found between the EG and CG regarding body weight and the weight of adrenal glands.

### Food, water and energy intake

**Figure 3:**
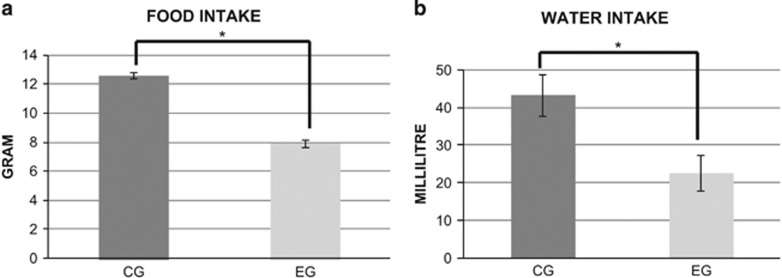
Food intake (**a**) and water intake (**b**) per day by animal during 15 weeks between the CG and EG expressed as grams (**a**) and millilitres (**b**). Asterisk indicates statistical difference between CG and EG. Results are expressed as mean±s.e.m.

Food and water intake of both groups are shown in Table 2 and Figure 3. Both food and water intake in the EG were lower than in the CG. There was no difference between the groups in terms of energy intake.

## Discussion

The model of diet used in our study caused metabolic changes, but it did not increase body weight. To the best of our knowledge, this is the first study designed to create an animal model diet containing lard and shortening, both saturated fatty acids, which are the most common types of fat found in processed food. Forty-five percent of the energy contained in this diet was supplied by fat compared with 4.5% in the control diet. The metabolic changes confirmed that the quality of the diet composition can have a direct influence on these markers.^28, 29^

Body weight was not correlated with the amount of VAT. In our study, we demonstrated that there is a non-causal relationship between these variables. Controversy surrounds the results from other studies showing an increase in body weight on an HFD. Unlike our study, which contained shortening and lard, Sampey *et al.*^30^ fed Wistar rats a lard-based 45% fat diet for 15 weeks causing higher body weight. It may be that the variety of ingredients used in manipulated HFDs and their flavours are important to determine weight gain. Also, it is a reason for concern that, in spite of the fact that the workers' body mass index is within normal limits, they may have increased visceral adiposity, which is an important risk factor for cardiovascular disease.

In disagreement with other studies, the animals of our study that were fed the HFD did not show hyperphagia because there was lower food intake, thus keeping their body weight and energy intake similar to those in the CG.^11, 31^ EG's lower food intake may have been due to an HFD with higher caloric density or less palatability. According to Erlanson-Albertsson,^32^ an HFD can upregulate the expression of hunger and satiety signals and, at the same time, blunt the response to satiety signals. Conversely, it has been reported that rats fed a higher calorie diet required spontaneously increased basal energy expenditure, stimulating thermogenesis as a compensatory mechanism in an attempt to maintain body weight.^33, 34^ In contrast to another study using streptozotocin combined with an HFD to change metabolic parameters and induce obesity and diabetes, hyperglycaemia was induced only by the HFD, which makes this model more similar to the natural course of disturbed metabolic physiopathology.^35^

In our research, the latency period for the development of metabolic changes was 15 weeks because of our attempt to trigger the most natural response in established parameters. This longer period allows a longer exposure to this type of food, as seen in shift workers. The changes in our sample were similar to those seen in shift workers after years of shift work. Shift workers have increased serum TG and serum total cholesterol in an intraindividual comparison.^36^ In terms of waist circumference, shift workers showed more central adiposity than day workers. This risk is increased in those who work night shifts for many years, including visceral fat area.^3, 37, 38, 39^ Night workers have higher risk to develop diabetes, and in a study with women working night shifts, they showed increased risk to develop type 2 diabetes in 3 years, and the risk gets higher as the working years extend.^40, 41^

Our study has some limitations. We did not weigh the animals' muscles. Therefore, this could be a potential confounding variable. According to the AIN-93M diet,^42^ the quantity of protein necessary for long-term studies to support proper growth and its maintenance is 12% of the energy intake. In the present study, the EG received 10% of energy intake, thus it is unlikely that the animals of this group had muscles' atrophy. Furthermore, the experimental diet was intended to mimic the night workers' diet because night workers eat a lower amount of proteins when compared with day workers. They also consume smaller amounts of some vitamins and minerals than day workers, such as vitamin A, calcium and selenium.^43^ No mineral mixture was added to the HFD. Therefore, there might have been loss of minerals' quantity in the HFD, but unfortunately, we did not measure the quantity of micronutrients present in lard and shortening. Also, we cannot assume that physical activity had a role in weight gain because it was not measured.

This study shows that feeding an HFD to experimental animals for a sustained period can lead to changes in metabolic parameters. Nevertheless, there was no significant increase in body weight although there was an increase in VAT. Body weight *per se* may not be a good predictor of metabolic change. Metabolic changes are related to the development of chronic diseases, such as a prediabetic state, type 2 diabetes, obesity and nonalcoholic fatty liver disease. In addition, because meal composition may be involved in the genesis of these diseases, studies using HFD can contribute to a better understanding of the metabolic physiopathology. Our results are robust as no difference was observed between the CG and EG at baseline, indicating that the animals were a homogeneous group at the beginning of the study.

In conclusion, the experimental diet was effective in showing that there were changes in serum glucose, TG and HDL-cho levels, as well as fat in the visceral tissue in spite of no change in body weight in 15 weeks.

## Figures and Tables

**Table 1:** Content of fatty acids in the diet

	*CG*	*EG*
*Saturated fatty acids (SFA)*		
Lauric acid (12:0)	0.10	0.04
Myristic acid (14:0)	1.36	1.13
Palmitic acid (16:0)	22.23	19.34
Stearic acid (18:0)	8.61	8.68
Arachidic acid (20:0)		0.08
Total%	32.3	29.27
		
*Unsaturated fatty acids*		
Palmitoleic acid (16:1)		0.91
Oleic acid (18:1)	35.91	39.45
Linoleic acid (18:2)	31.69	20.65
Linolenic acid (18:3)	0.10	0.59
MUFA%	36.01	40.96
PUFA%	31.69	20.65
Total%	67.7	61.6

Abbreviations: CG, control group; EG, experimental group; MUFA, monounsaturated fatty acid; PUFA, polyunsaturated fatty acid. Amounts expressed as percentage (%) in 100 g of diet.

**Table 2:** Characteristics of the sample at baseline and after 15 weeks of a high-fat diet intervention

	*CG (*n*=9)*	*EG (*n*=10)*	T	P
*Baseline*				
Body weight (g)	254.23 (±6.89)	246.66 (±7.76)	0.72	0.47
Serum glucose (mg dl^−1^)	152.40 (±4.11)	166.30 (±7.13)	−1.68	0.11
HDL-cholesterol (mg dl^−1^)	49.60 (±1.40)	47.20 (±1.32)	1.24	0.23
Triglycerides (mg dl^−1^)	88.40 (±8.68)	108.00 (±9.21)	−1.54	0.14
				
*After 15 weeks*				
Body weight (g)	402.73 (±13.48)	391.24 (±19.08)	0.48	0.63
Visceral adipose tissue (g)	17.12 (±1.76)	34.22 (±4.70)	−3.26	0.005**
Liver weight (g)	11.33 (±1.50)	13.17 (±0.52)	2.62	0.01**
Adrenal gland weight (g)	0.05 (±0.005)	0.07 (±0.005)	1.89	0.07
Food intake (g per day per rat)	12.6 (±0.21)	7.9 (±0.23)	24.5	<0.001***
Water intake (ml per day per rat)	43.3 (±5.64)	22.6 (±4.71)	22.23	<0.001***
Energy intake (Kcal per day per rat)	50.6 (±0.65)	49.4 (±1.49)	0.70	0.48

Abbreviations: CG, control group; EG, experimental group; HDL, high-density lipoprotein. Data presented as mean (±s.e.m.). Student's *t*-test for two independent samples.

Significant *P*-values: *P<0.05; **P<0.01; ***P<0.001.
